# The *Pseudomonas aeruginosa* Reference Strain PA14 Displays Increased Virulence Due to a Mutation in *ladS*

**DOI:** 10.1371/journal.pone.0029113

**Published:** 2011-12-22

**Authors:** Helga Mikkelsen, Rachel McMullan, Alain Filloux

**Affiliations:** Division of Cell and Molecular Biology, Department of Life Sciences, Imperial College London, London, United Kingdom; Vrije Universiteit Brussel, Belgium

## Abstract

*Pseudomonas aeruginosa* is a pathogen that causes acute and chronic infections in a variety of hosts. The pathogenic potential of *P. aeruginosa* is strain-dependent. PA14 is a highly virulent strain that causes disease in a wide range of organisms, whereas PAO1 is moderately virulent. Although PA14 carries pathogenicity islands that are absent in PAO1, the presence or absence of specific gene clusters is not predictive of virulence. Here, we show that the virulent strain PA14 has an acquired mutation in the *ladS* gene. This mutation has a deleterious impact on biofilm, while it results in elevated type III secretion system (T3SS) activity and increased cytotoxicity towards mammalian cells. These phenotypes can be reverted by repairing the *ladS* mutation on the PA14 genome. The RetS/LadS/GacS signaling cascade is associated with virulence and the switch between acute and chronic infections. RetS is a sensor that down-regulates biofilm formation and up-regulates the T3SS. Mutations in *retS* are acquired in strains isolated from chronically infected cystic fibrosis patients and lead to hyperbiofilm formation and reduced cytotoxicity. Conversely, the LadS sensor promotes biofilm formation and represses the T3SS. We conclude that the *ladS* mutation is partly responsible for the high cytotoxicity of PA14, and our findings corroborate the central role of RetS and LadS in the switch between acute and chronic infections. Given the extensive use of the reference strain PA14 in infection and virulence models, the bias caused by the *ladS* mutation on the observed phenotypes will be crucial to consider in future research.

## Introduction

*Pseudomonas aeruginosa* is a Gram-negative bacterium that thrives in a wide range of natural environments, both terrestrial and aquatic. It is also well known as an opportunistic pathogen with a broad host range that includes humans. This versatility is reflected in a relatively large and complex genome, which ranges from 5.1 to 7 Mb in size [1]. The first *P. aeruginosa* genome to be sequenced was that of strain PAO1 [2], and one of the striking features was the high proportion of coding capacity dedicated to regulation (around 10% of the predicted open reading frames). Unlike strict human pathogens, selection has therefore favored complexity rather than specialization in the genome of this species.

*P. aeruginosa* isolates display a striking variability in virulence, ranging from very moderate to highly virulent strains [3], [4], [5]. PAO1, which was isolated from a wound over 50 years ago, is a moderately virulent strain [6] and belongs to a relatively rare clonal group. In contrast, the clinical strain PA14 is a highly virulent isolate and represents the most common clonal group worldwide [1]. The genome of PA14 was published in 2004 and revealed a high degree of conservation compared to PAO1 [7]. However, the PA14 genome also contains two pathogenicity islands that are absent in PAO1, and these carry several genes implicated in virulence. Examples of this are *exoU*, which encodes an effector of the type III secretion system (T3SS), and the Rcs/Pvr two-component systems that control the *cupD* fimbrial cluster [7], [8], [9]. A recent study has further shown that deletion of one or both pathogenicity islands impacts the virulence of PA14 in murine models of acute pneumonia and bacteremia [10]. Despite this, a study of a series of *P. aeruginosa* isolates failed to identify any correlation between PA14-specific genes and the level of virulence [6]. The presence of specific genes or clusters may therefore not be predictive of virulence in a given strain.

A large number of infection models have been employed to assess the pathogenicity of *P. aeruginosa* during both acute and chronic infection. The variety of potential hosts is a further confirmation of the extraordinary versatility of the pathogen. In addition to mammalian systems, the models include plants (lettuce leaves and *Arabidopsis thaliana*), invertebrates (*Caenorhabditis elegans*), insects (*Galleria mellonella* and *Drosophila melanogaster*) and vertebrates (zebrafish) [11], [12], [13]. Although PAO1 is infectious in some of these models, the majority of them employ PA14, which displays higher virulence in most hosts [5], [14], [15].

Some studies have used transposon mutant screens to identify functions that are required during infection in a certain host [15], [16], [17], [18], [19]. These screens have linked a variety of functions to virulence, such as chemotaxis, quorum sensing, transcriptional regulators, multidrug transporter or phenazine production. Interestingly, some factors appear to be required for full virulence in a series of different hosts, suggesting that they may play a more universal role during infection. One of these systems is the GacS/GacA two-component system, and mutants in the *gacS/gacA* genes are attenuated for virulence in plants, nematodes, insects or mouse models of infection [4], [15], [20], [21].

Of the over 60 two-component systems encoded on the *P. aeruginosa* genome [22], the Gac system therefore appears to play a unique role. The GacS sensor phosphorylates the GacA response regulator, which promotes transcription of only two genes, namely the small RNAs (sRNAs) RsmY and RsmZ. These sRNAs relieve the repression exerted by the post-transcriptional repressor RsmA on genes that are known to be required for biofilm formation, such as the *pel* and *psl* genes [23], [24], [25]. Furthermore, the activity of the GacS/GacA two-component system is inversely regulated by two additional sensors, RetS and LadS [26], [27]. RetS inhibits the activity of GacS by forming inactive heterodimers, and this promotes motility and cytotoxicity [28]. Conversely, LadS activates the GacS/GacA cascade *via* an unknown mechanism [27], and this suppresses cytotoxicity while promoting biofilm formation.

In this study, we show that the PA14 reference strain has a mutation in the *ladS* gene, which leaves the protein out of frame. This mutation results in repression of biofilm formation and enhanced production of the T3SS, leading to elevated cytotoxicity in this strain.

## Results

### *P. aeruginosa* PA14 has a mutation in the *ladS* gene

**Figure 1:**
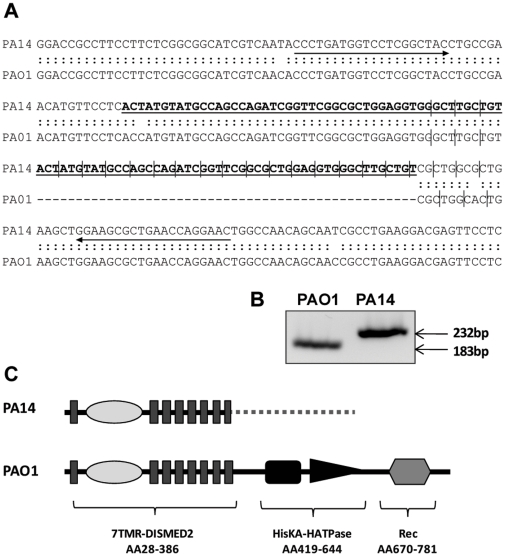
PA14 is a *ladS* mutant. (A) Alignment of the *ladS* open reading frame of PAO1 and PA14 starting at +1021 bp. The bold underlined sequence in PA14 shows the 49 bp duplication, which alters the PA14 reading frame compared to PAO1, illustrated by vertical lines. PCR primers are indicated by arrows. (B) PCR products of the genomic region indicated in (A), separated by agarose gel electrophoresis. (C) Predicted domain organization of LadS in PA14 and PAO1. Nonsense protein sequence in PA14 is shown with a dotted line.

The genomic region encoding the LadS sensor is conserved across several *Pseudomonas* species. LadS is a hybrid sensor kinase with an N-terminal 7TMR-DISMED2 sensing domain, which is followed by a histidine kinase domain and a receiver domain. However, an analysis of the PA14 genome [29] revealed that the *ladS* open reading frame had not been annotated (http://www.pseudomonas.com) [30], even though the flanking regions appeared to be conserved. A detailed investigation of the DNA sequence (located between 1069280–1071716 nucleotide position on the genome) showed that the sequence was indeed conserved (Figure 1A), but that a 49-nucleotide duplication had occurred in the coding sequence, leading to a frame shift and a gene encoding a putative truncated protein. The predicted product of the *ladS* gene in PA14 has the sensing domain intact, but the gene is out of frame from codon number 380. From there, a new frame is kept for an additional 202 codons, resulting in a 581 amino acids-long protein, with an aberrant C terminus (Figure 1C and Figure S1 panel A). This altered C-terminus did not display any predicted domain structure or similarity to other known proteins. We confirmed that the duplication was indeed present in PA14 by designing primers flanking the insertion site that yielded a 183 bp product in PAO1 and 232 bp in PA14 (Figure 1B), which suggests that LadS is not functional in this isolate. The recently updated *Pseudomonas* website (http://www.pseudomonas.com) includes four incomplete *P. aeruginosa* genomes in addition to the existing four complete sequences. Apart from PA14, these strains all have an intact *ladS* gene resulting in a highly conserved predicted protein sequence (Figure S1 panel B). We further investigated whether the PA14 mutation was present in other *P. aeruginosa* strains by PCR amplifying the relevant region in four previously published environmental isolates [6]. However, these strains all appeared to have an intact *ladS* gene (Figure S2).

### Reconstruction of the *ladS* gene in PA14 influences biofilm formation

Apart from the 49 extra nucleotides, the sequence of *ladS* is highly conserved between PAO1 and PA14 (Figure 1A). In fact, if the insertion was to be removed, the resulting PA14 LadS protein would only have one seemingly neutral amino acid change (V509A) compared to PAO1. We therefore removed the duplication by generating a mutator fragment comprising around 1200 bases of *ladS* sequence amplified from PAO1, which was cloned into the suicide vector pKNG101. After double crossover on the PA14 genome, the correction was verified by PCR and sequencing, generating the strain *ladS*_R_. This strain displayed the same growth rate as PA14 in all conditions tested.

**Figure 2:**
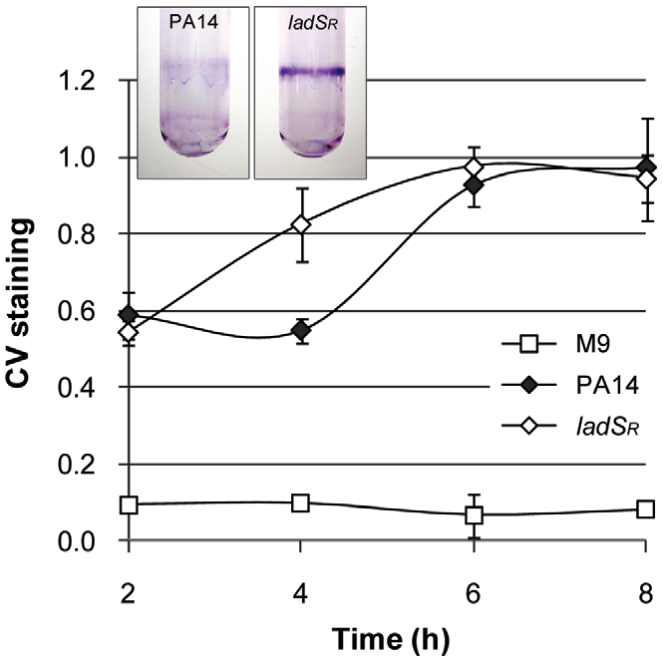
Biofilm formation. Biofilm formation of PA14 and *ladS*_R_ in M9 medium. Graph: Biofilm formation in 24-well polystyrene plates at 37°C in shaking conditions. Inset: Crystal violet stained biofilms in glass tubes after 8 h incubation at 30°C. Data is representative of at least two independent experiments.

LadS was originally identified in *P. aeruginosa* PAK as a “**L**ost **ad**herence **S**ensor” [27]. Indeed, a mutation in the *ladS* gene resulted in a strain that was unable to form biofilms due to down-regulation of genes involved in Pel polysaccharide biogenesis. We therefore tested the ability of PA14-*ladS*_R_ to form biofilms in microtiter plates incubated at 37°C with shaking and showed that the *ladS*_R_ strain displayed accelerated biofilm formation compared to PA14 (Figure 2). This delayed attachment phenotype was verified in glass tubes incubated at 30°C without shaking (Figure 2, inset). The acquired *ladS* mutation in PA14 therefore slows down biofilm formation in this strain, and this can be corrected by restoring the original frame of the gene.

### LadS up-regulates the *pel* and T6SS genes and down-regulates the T3SS genes

The RetS/LadS/GacS signaling network converges on the activity of the post-transcriptional regulator RsmA, the specific action of which is to bind to and prevent translation of target mRNAs [23], [31], [32]. Apart from *ladS* in PA14, the components of this signaling cascade are all conserved in the completely sequenced strains of *P. aeruginosa* (http://www.pseudomonas.com). Among the mRNAs that are repressed by RsmA are the Pel and Psl polysaccharides as well as the T6SS (H1-T6SS) [23], which has been shown to play an important role in chronic infection [33], [34]. Furthermore, RsmA is known to enhance the activity of the T3SS, which is involved in cytotoxicity and acute infection, although the molecular mechanisms of this are currently unknown. In this context, it is important to note that PA14 displays strain-specific differences, both with respect to polysaccharide production and the T3SS. While Psl is the primary polysaccharide in PAO1, Pel plays a more important role in PA14, which does not have a complete *psl* gene cluster [35]. Furthermore, the PA14 genome does not encode the type III effector ExoS, but instead carries the gene encoding the potent cytotoxin ExoU on the PAPI-2 pathogenicity island [7].

**Figure 3:**
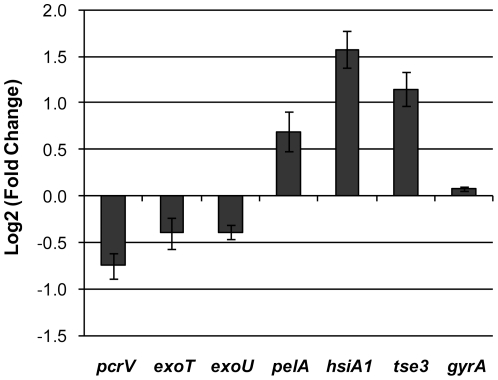
qRT-PCR analysis of target genes in *ladS*_R_ compared to PA14. Positive values indicate that mRNA levels are higher in *ladS*_R_. All mRNA levels have been normalized to 16S rRNA.

In order to assess the impact of the *ladS* mutation on target genes, a qRT-PCR analysis of relevant mRNAs involved in polysaccharide production, T3S and T6S was carried out on cells harvested in exponential phase. In these conditions, the *pelA* transcript displayed a 60% increase in the *ladS*_R_ strain compared to the PA14 (Figure 3). Furthermore, *pcrV* (T3SS machinery) *exoT* and *exoU* (T3SS effectors) were down-regulated 30–60% compared to the PA14 wild type. Conversely, *hsiA1*, which is the first gene in the H1-T6SS gene cluster [36], and the T6SS substrate *tse3* [37], displayed a 3 and 2-fold increase respectively (Figure 3). Restoration of the *ladS* gene in PA14 therefore results in a modification in the expression profile of the RsmA targets. This suggests that despite the *ladS* mutation in PA14, the rest of the Ret/Gac/Rsm cascade appears fully functional and still able to respond to LadS signaling.

### Restoration of the *ladS* gene switches the activity of the T3SS and T6SS

**Figure 4:**
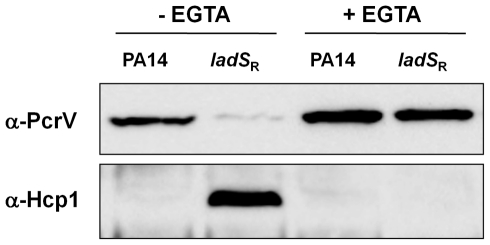
Immunoblots of PA14 and *ladS*_R_ cell lysates using antibodies directed against structural components of the T3SS and T6SS (PcrV and Hcp1 respectively). The calcium chelator EGTA was added to the growth medium to induce the T3SS as indicated.

Since the changes observed at the mRNA level were relatively modest, especially for the T3SS, we investigated whether any differences could be observed at the protein level. Western blotting was carried out using antibodies directed against structural components of the T3SS [38] and T6SS [39] (PcrV and Hcp1, respectively). This showed that *ladS*_R_ produced markedly reduced levels of PcrV compared to PA14 in standard conditions (Figure 4). We then investigated whether the T3SS could be induced in the *ladS*_R_ strain to the same extent as in the wild type by adding the chelating agent EGTA to the growth medium. In these conditions, both strains displayed increased and similar levels of PcrV, suggesting that induction of the T3SS by EGTA overrules the repressing effect of LadS.

In order to investigate whether this regulation of the T3SS was mirrored in the expression of the T6SS, we carried out Western blots using an antibody directed against the putative structural component Hcp1 [39]. Previous studies have shown that the T6SS is not expressed in *P. aeruginosa* PAK wild type, but that it is activated in PAKΔ*retS* [26]. In agreement with this, we were unable to detect Hcp1 in PA14 wild type. However, in the *ladS*_R_ strain there was a strong induction of the T6SS (Figure 4), which clearly demonstrates the effect of LadS in the regulation of this secretion system. It may seem curious that two strains with an intact *ladS* gene (PAK wild type and PA14-*ladS*_R_) display very different basal levels of Hcp1. However, studies in our lab have shown that the production of the T6SS varies substantially between strains, and this is also known to be the case for the T3SS. Interestingly, Hcp1 could not be detected in the *ladS*_R_ strain in inducing conditions for the T3SS. This observation highlights the antagonistic regulation of the T3SS and T6SS. It is also worth noting that the restoration of LadS appeared to have a more pronounced influence on protein levels than on mRNA levels. This could be explained by the post-transcriptional activity of RsmA.

### Restoration of the *ladS* gene reduces cytotoxicity towards mammalian cells

**Figure 5:**
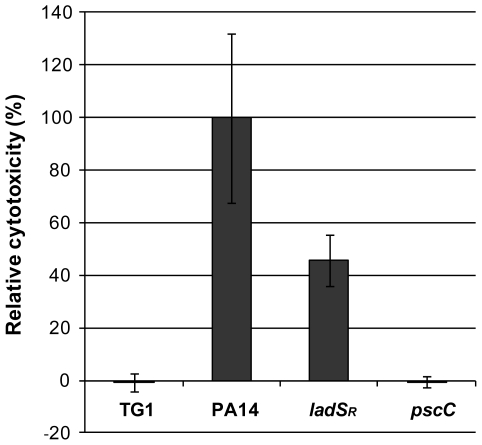
Lactate dehydrogenase (LDH) release of HeLa cells infected with the indicated bacterial strains for 1 hour. The LDH release of PA14 has been set to 100%. Data are representative of three independent experiments. The difference between PA14 and *ladS*_R_ is significant with p = 0.018.

The shift in secretion profile due to the mutation in *ladS* could have important implications for the behavior of PA14 during infection. We therefore tested the cytotoxicity of PA14 and *ladS*_R_ in two different models of infection. As previously mentioned, a PA14::Tn-*gacA* mutant is attenuated in the commonly used *C. elegans* slow killing assay [12], [17] [40]. However, restoration of *ladS* did not alter the *C. elegans* killing pattern, which was identical to that of the reference strain (Figure S3). Cytotoxicity was then monitored by lactate dehydrogenase (LDH) release in HeLa cells. After one hour of infection, *ladS*_R_ only displayed around 50% of the LDH release observed in PA14 (Figure 5). This decrease in cytotoxicity was likely due to the reduced activity of the T3SS, since a PA14Tn::*pscC* transposon mutant [41], which is defective in T3S, did not display any detectable cytotoxicity in these conditions. The LDH release in the *pscC* mutant was comparable to the *E. coli* TG1 negative control. These data clearly demonstrate that the high virulence of *P. aeruginosa* PA14 can be at least partly attributed to a hyperactive T3SS due to the absence of a functional LadS sensor.

## Discussion

*P. aeruginosa* is an environmental bacterium that thrives in a variety of habitats and is a successful colonizer of a broad range of hosts, including humans. This unique ability to cope with diverse environments has been attributed to the large and plastic genome [42]. Horizontal gene transfer plays an important role in acquisition of new traits, but is not necessarily accompanied with the loss of other genes. PA14 carries two pathogenicity islands that are not present in PAO1 [7]. Intuitively, this could explain the marked virulence difference between these two strains, but this seems not to be the case. Indeed, although mutations in PA14 specific genes have been found to attenuate virulence of the strain [6], [7], the presence of the same genes in other isolates could not be correlated with increased virulence [6]. If the presence of a specific subset of genes alone cannot account for the virulence degree of a particular isolate, variation in transcription and hypermutability may be critical features for adaptation/evolution in the course of an infection [43], [44], [45]. A number of studies have suggested that microevolution of *P. aeruginosa* in cystic fibrosis patients is dynamic and results in a large population diversity with an adaptation, but not necessarily a loss, of their virulence traits [46], [47], [48]. Besides LasR and other components of the quorum sensing systems, the RetS/LadS/GacS signaling network, which is conserved in most Pseudomonas species, has been extensively linked to pathogenesis [49], [50]. Mutants in the central two-component system GacS/GacA are attenuated in multiple models of infection [4], [20], [21], [51], suggesting a general role in virulence. The GacA response regulator has only two known targets, namely the sRNAs RsmY and RsmZ [28]. These RNAs sequester the translational repressor RsmA, which has proven to be crucial in the switch between cytotoxicity and biofilm formation, as well as between chronic and acute infections. Direct targets of RsmA repression include the *pel* and *psl* genes involved in biofilm formation, and the T6SS, which is important during chronic infection. Conversely, the T3SS is positively regulated by RsmA, probably in an indirect manner [23]. The activity of the Gac/Rsm signaling cascade is inversely controlled by the orphan sensors RetS and LadS [26], [27]. A *retS* mutant is a hyperbiofilm former that displays low cytotoxicity, while a *ladS* mutant displays low levels of biofilm formation and enhanced cytotoxicity. The significance of this regulatory network is further confirmed by the recent identification of key adaptive mutations in the *retS* gene during chronic infection of CF airways [52]. A *retS* mutant displays a hyperbiofilm phenotype and a reduced cytotoxicity [26]. In this particular case, it seems that a reduction of virulence of the strain could be associated with adaptation to chronic infection in CF patients.

In this study, we show that the hyper-virulent strain PA14 is a *ladS* mutant. The observed duplication in the *ladS* open reading frame may be the result of DNA repair following a double stranded break, which leaves the protein truncated and out of frame. This is likely to be a relatively recent event, given the absence of secondary mutations in the gene. The impact of the mutation was evaluated by restoring the native gene on the chromosome, generating *ladS*_R_. The restored strain displayed enhanced biofilm formation, increased expression of the T6SS, and reduced expression of the T3SS. Importantly, expression of the T3SS effector coded by the *exoU* gene that is located on the pathogenicity island PAPI-2 is controlled by the core genome-encoded LadS sensor. These changes were observed at the transcriptional, as well as on the protein or phenotypic level. Furthermore, the change in the T3SS translated into reduced cytotoxicity, as evaluated by LDH release in HeLa cells. Conversely, no difference was observed in a *C. elegans* slow killing assay, which has been shown to be independent of the T3SS [53].

Mutations leading to reduced virulence are more commonly reported than mutations leading to hypervirulence. This could be partly because many infection models have primarily been developed to screen for attenuation of virulence. The identification of a *ladS* mutation in the hyper-virulent burn wound isolate PA14, combined with the fact that it is the only sequenced *P. aeruginosa* strain that lacks a functional *psl* polysaccharide biosynthetic cluster [54], suggest that PA14 behavior during infection may not be representative of other members of the species. Given that the vast majority of virulence and cytotoxicity studies have been carried out using either PA14 or PAO1, it is crucial to take into account that the PA14 isolate is a *ladS* mutant and that this mutation has marked consequences on its pathogenesis.

## Materials and Methods

### Strains and growth conditions

Unless otherwise stated, cultures were grown in LB at 37°C with shaking. Primers used in this study are listed in Table S1. The *ladS* duplication in PA14 was verified using primers ladSF and ladSR. The PA14-*ladS*_R_ strain was generated as follows. A 1 200 bp region of the *ladS* gene was amplified by PCR from PAO1 genomic DNA using primers ladS1 and ladS2. The product was cloned into pCR2.1-TA and sequenced, then sub-cloned into the *P. aeruginosa* suicide vector pKNG101. After sucrose selection, restored clones were identified using primers ladSF and ladSR, and the crossover was confirmed by sequencing of the 1 200 bp mutator region.

### Biofilm formation

Attachment assays were carried out in 24-well polystyrene plates. Overnight cultures were inoculated into M9 medium with glucose to a final O.D_600 nm_ of 0.1 and incubated at 37°C with shaking. Attached cells were stained with 0.1% crystal violet (CV) at the indicated time points. Unbound dye was removed by washing twice with water, the CV was dissolved in 96% ethanol and absorbance was monitored at 600 nm. Visualization of biofilms was carried out in M9 medium in glass tubes that were incubated at 30°C without shaking and stained with crystal violet as described above.

#### qRT-PCR

Overnight cultures were sub-cultured into LB, grown to exponential phase (O.D_600 nm_ 1) and harvested into RNAlater (Ambion). RNA extraction, reverse transcription and qPCR was carried out as previously described [55]. Gene expression was normalized to 16S rRNA.

### Immunoblotting

For Western analysis, cultures were inoculated into LB medium to O.D_600 nm_ 0.1 and incubated at 37°C with shaking for 5 hours. Induction of the T3SS was achieved by adding EGTA (5 mM) and MgCl_2_ (20 mM) to the growth medium. Whole cell lysates were separated by SDS-PAGE. Primary antibodies, α-Hcp1 [56] and α-PcrV (kindly provided by A. Rietsch) were used at dilutions of 1∶5 000 and 1∶10 000 respectively. Secondary antibody (horseradish peroxidise-conjugated goat anti-rabbit IgG, Sigma) was used at 1∶5 000 dilution. Visualization was achieved using the SuperSignal West Pico Chemiluminescent Substrate Kit (Thermo) and a LAS3000 Imaging System (Fuji).

### Lactate dehydrogenase (LDH) release assay

The cytotoxicity of PA14 and PA14-*ladS*_R_ was assessed using HeLa cells (ATCC CCL-2) using *E. coli* TG1 and PA14Tn::*pscC* (PA14Tn::PA14_42350) [41], which is defective in T3S, as negative controls. HeLa cells were routinely maintained in Dulbecco's Modified Eagle's Medium with 1 000 mg/L glucose (Sigma), supplemented with FBS, non-essential amino acids and GlutaMAX (Invitrogen). Cytotoxicity assays were carried out on confluent cells in 96-well plates. Prior to infection, cells were washed twice in PBS, and the medium was changed to RPMI 1640 without phenol red (Invitrogen). Bacterial strains were sub-cultured from overnight cultures into LB to a final OD_600 nm_ of 0.1 and grown at 37°C with shaking for 2 hours. Cells were then harvested, washed twice in PBS and resuspended in RPMI 1640 medium. HeLa cells were infected with an initial multiplicity of infection of 20, and the infection was synchronized by centrifugation for 5 minutes at 1 000 *g*. After incubation for 1 hour at 37°C in 5% CO_2_, LDH release was assayed using the CytoTox 96 Assay Kit (Promega) following the supplier's instructions.

## Supporting Information

Figure S1 Alignment of predicted protein sequences of LadS in various strains of *P. aeruginosa* (MultAlin, Corpet *et al.* 1988). A) PAO1 *vs.* PA14. B) Seven *P. aeruginosa* strains available in the Pseudomonas database (Winsor *et al.* 2011). **Supplementary reference** Corpet F (1988): Multiple sequence alignment with hierarchical clustering. Nucl. Acids Res. 16(22), 10881–10890.
(PPTX)

Figure S2 PCR of a section of the *ladS* gene in PAO1, PA14 and four environmental isolates (Lee *et al.* 2006) as indicated using primers ladSF and ladSR (Table S1).
(PPTX)

Figure S3 *C. elegans* slow killing assay. L4 larvae were transferred onto lawns of PA14 or *ladS_R_* as previously described (Powell & Ausubel, 2008), and viability was scored at the indicated time points. Data is representative of three independent experiments.
(PPTX)

Table S1 Oligonucleotides used in this study.
(DOCX)
