# Supplementary figures and images for: The Pseudomonas aeruginosa Reference Strain PA14 Displays Increased Virulence Due to a Mutation in ladS
Source: PLoS One. 2011 Dec 22;6(12):e29113. doi: 10.1371/journal.pone.0029113 (PMC3245244; doi:10.1371/journal.pone.0029113)

## Slide 1
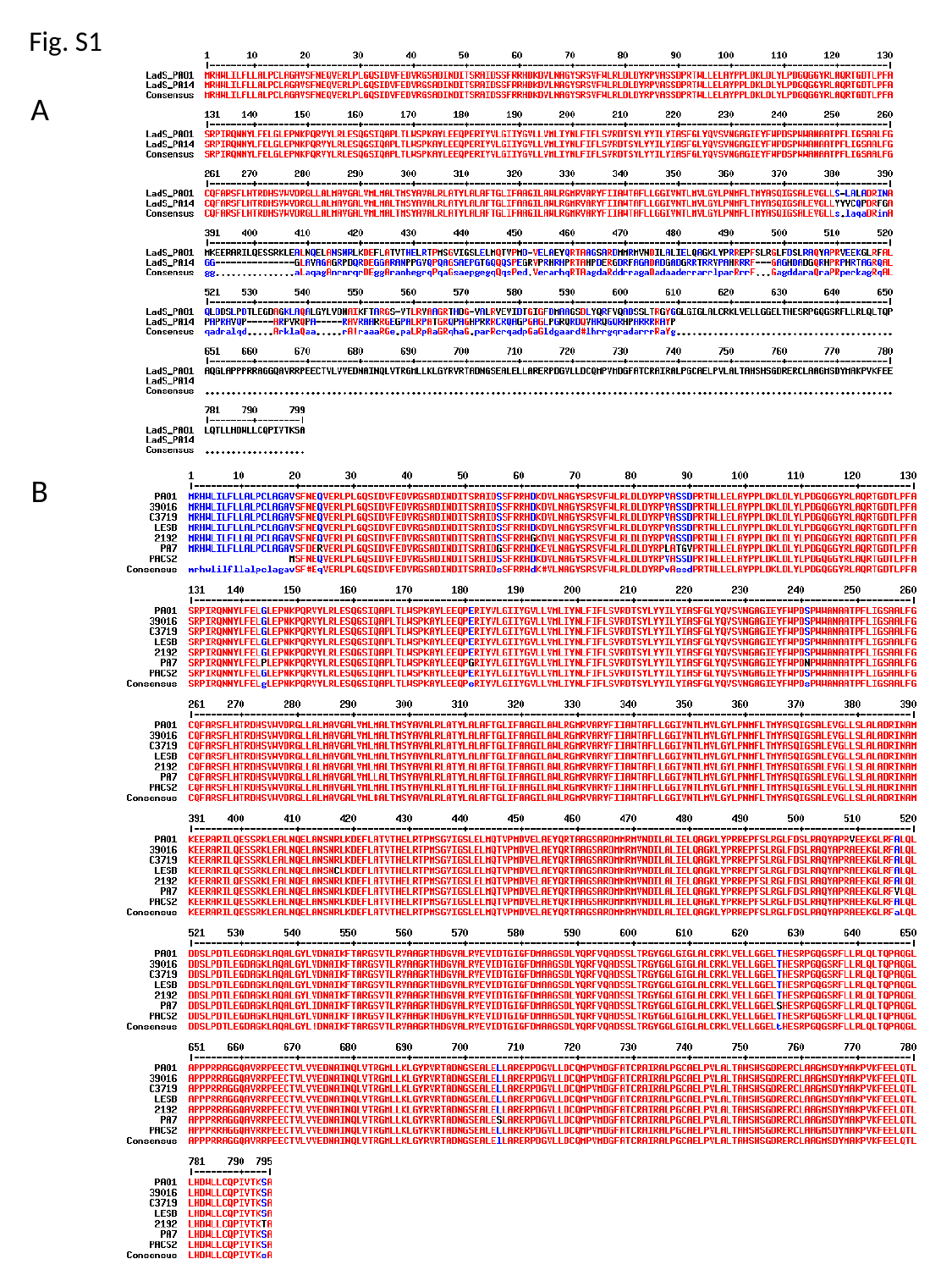

Fig. S1
A
B

Supplement: Figure S1 — Alignment of predicted protein sequences of LadS in various strains of P. aeruginosa (MultAlin, Corpet et al. 1988). A) PAO1 vs. PA14. B) Seven P. aeruginosa strains available in the Pseudomonas database (Winsor et al. 2011). Supplementary reference Corpet F (1988): Multiple sequence alignment with hierarchical clustering. Nucl. Acids Res. 16(22), 10881–10890. (PPTX) [file pone.0029113.s001.pptx]

## Slide 1
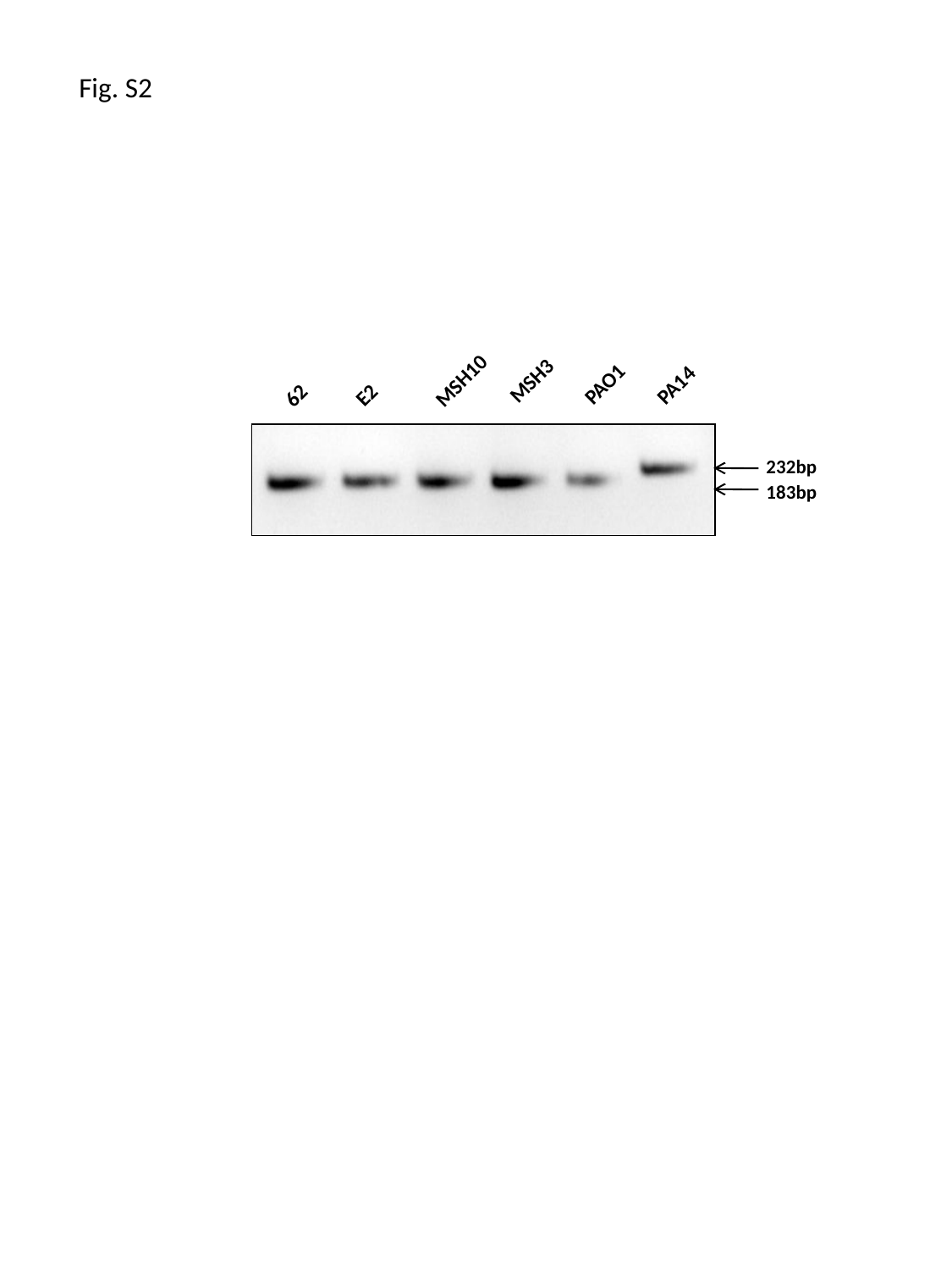

Fig. S2
MSH10
MSH3
PAO1
PA14
E2
62
232bp
183bp

Supplement: Figure S2 — PCR of a section of the ladS gene in PAO1, PA14 and four environmental isolates (Lee et al. 2006) as indicated using primers ladSF and ladSR (Table S1). (PPTX) [file pone.0029113.s002.pptx]

## Slide 1
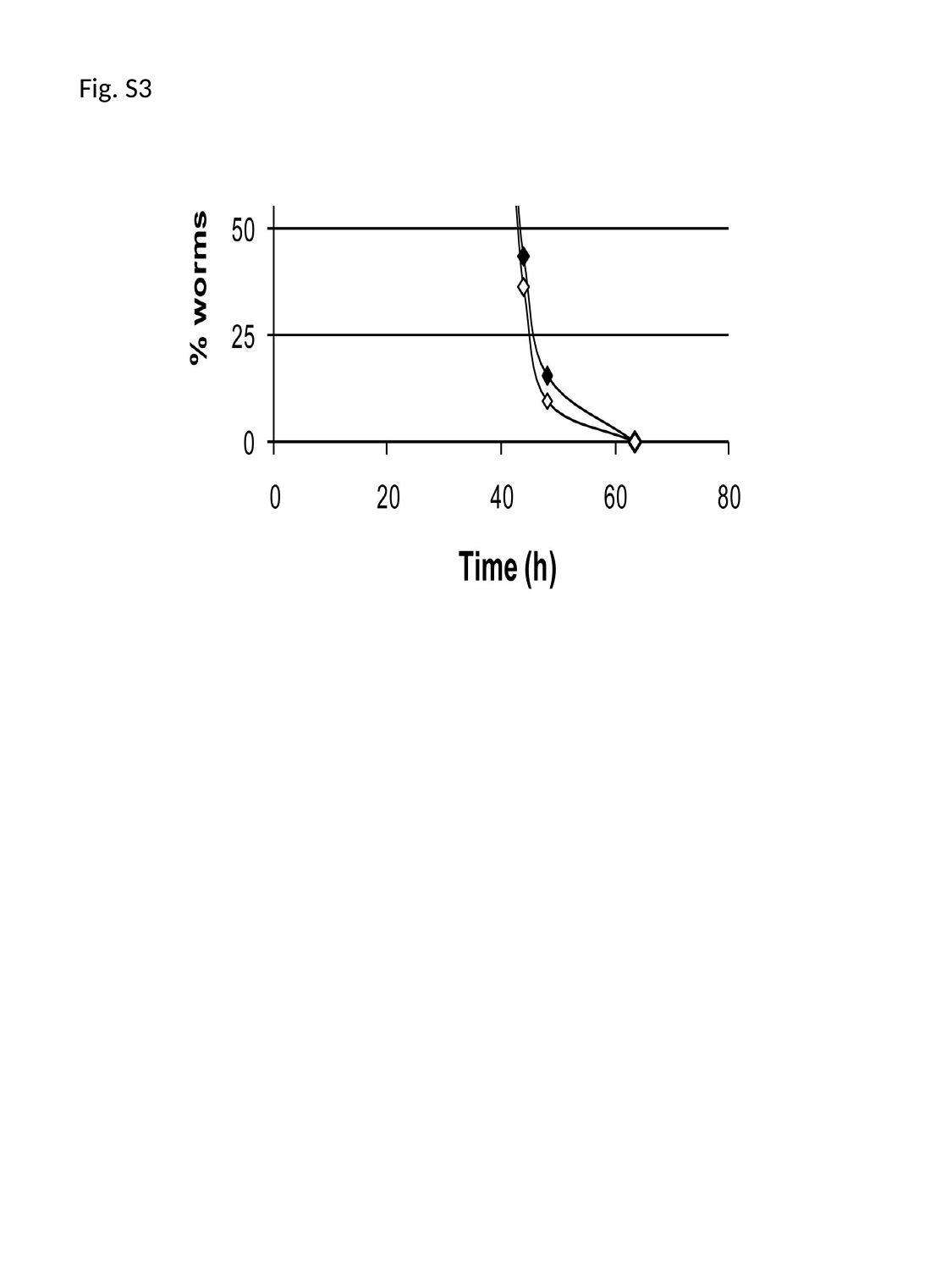

Fig. S3

Supplement: Figure S3 — C. elegans slow killing assay. L4 larvae were transferred onto lawns of PA14 or ladSR as previously described (Powell & Ausubel, 2008), and viability was scored at the indicated time points. Data is representative of three independent experiments. (PPTX) [file pone.0029113.s003.pptx]
