# Supplementary material for: The Pseudomonas aeruginosa Reference Strain PA14 Displays Increased Virulence Due to a Mutation in ladS
Source: PLoS One. 2011 Dec 22;6(12):e29113. doi: 10.1371/journal.pone.0029113 (PMC3245244; doi:10.1371/journal.pone.0029113)
Supplement: Table S1 — Oligonucleotides used in this study. (DOCX) [file pone.0029113.s004.docx]

**Table S1:** Oligonucleotides used in this study

| **Oligo name** | **Sequence** | **Use** | **Reference** |
| --- | --- | --- | --- |
| ladSF | CCCTGATGGTCCTCGGCTAC | Amplification of putative *ladS* mutation in PA14 | This study |
| ladSR | GTTCCTGGTTCAGCGCTTCC | Amplification of putative *ladS* mutation in PA14 | This study |
| ladS1 | AGCAGCCCGAACGCATCTAT | Amplification of *ladS* mutator from PAO1 | This study |
| ladS2 | CCGGTGTCGATCACTTCCAC | Amplification of *ladS* mutator from PAO1 | This study |
| 16S rRNAF | CGCCGTAAACGATGTCGACTA | qPCR primer | [52] |
| 16S rRNAR | TTAACCTTGCGGCCGTACTC | qPCR primer | [52] |
| pcrV F | AGTGGGATCTGCGCGAGTT | qPCR primer | This study |
| pcrV R | TGGGTCTGCAGGACATCCTT | qPCR primer | This study |
| exoT F | TCTGCCGCCGAGATCAAG | qPCR primer | This study |
| exoT R | GCCTCTCCGCTGTCAAAGTC | qPCR primer | This study |
| exoU F | TACCAGGCCGGCGTAGAGT | qPCR primer | This study |
| exoU R | GTGGCCCGCTGTCAAAATT | qPCR primer | This study |
| pelA F | CCTTCAGCCATCCGTTCTTCT | qPCR primer | This study |
| pelA R | TCGCGTACGAAGTCGACCTT | qPCR primer | This study |
| hsiA1 F | ATCGCCTGCTCGAGTATTACG | qPCR primer | This study |
| hsiA1 R | GGGATGAGATTCCTCACGATTT | qPCR primer | This study |
| tse3 F | GGCACGCAATGCCTTGAT | qPCR primer | This study |
| tse3 R | GCAGATGTCGAAGAAGGTGATG | qPCR primer | This study |
| gyrA F | AACGACTGGAACAAGCCCTACA | qPCR primer | This study |
| gyrA R | GCGCACGATGGTGTCGTA | qPCR primer | This study |
